# GFAP and antibodies against NMDA receptor subunit NR2 as biomarkers for acute cerebrovascular diseases

**DOI:** 10.1111/jcmm.12614

**Published:** 2015-06-17

**Authors:** Delia Maria Stanca, Ioan Constantin Mărginean, Olga Sorițău, Cristian Dragoș, Mariana Mărginean, Dafin Fior Mureșanu, Johannes C Vester, Alexandru Rafila

**Affiliations:** a Department of Neurosciences, “Iuliu Hatieganu” University of Medicine and Pharmacy Cluj-NapocaCluj-Napoca, Romania; b Department of Cancer Immunology of “Prof. dr. Ion Chiricuță” Oncologic Institute Cluj-NapocaCluj-Napoca, Romania; c Department of Statistics, Babes-Bolyai University Cluj-NapocaCluj-Napoca, Romania; d Department of Histology, “Iuliu Hatieganu” University of Medicine and Pharmacy Cluj-NapocaCluj-Napoca, Romania; e “RoNeuro” Institute for Neurological Research and DiagnosticCluj-Napoca, Romania; f Department of Biometry and Clinical Research, IDV Data Analysis and Study PlanningKrailling, Germany; g Department of Microbiology and Epidemiology, University of Medicine and Pharmacy “Carol Davila”Bucharest, Romania

**Keywords:** ischaemic stroke, intracerebral haemorrhage, GFAP, NMDA, neuronal biomarkers

## Abstract

We studied whether the serum levels of glial fibrillary acidic protein (GFAP) and of antibodies against the *N*-methyl-d-aspartate receptor subunit NR2 (NR2 R_NMDA_) can discriminate between intracerebral haemorrhage (ICH) and ischaemic stroke (IS) in stroke patients. We prospectively recruited patients with suspected stroke (72 confirmed) and 52 healthy controls. The type of brain lesion (ICH or IS) was established using brain imaging. The levels of GFAP and of antibodies against NR2 R_NMDA_ were measured in blood samples obtained within 12 hrs after stroke onset and 24, 48 and 72 hrs and 1 and 2 weeks later using ELISA immunoassay. Improvement in diagnostic performance was assessed in logistic regression models designed to predict the diagnosis and the type of stroke. GFAP peaks early during haemorrhagic brain lesions (at significantly higher levels), and late in ischaemic events, whereas antibodies against NR2 R_NMDA_ have significantly higher levels during IS at all time-points. Neither of the two biomarkers used on its own could sufficiently discriminate patients, but when they are used in combination they can differentiate at 12 hrs after stroke, between ischaemic and haemorrhagic stroke with a sensitivity and specificity of 94% and 91%, respectively.

## Introduction

Brain imaging is still the gold standard for differentiating the type of brain lesions in a stroke patient (ischaemia or haemorrhage) 1. Both the computerized tomography(CT) and the MRI scan of the brain require hospital admittance and lead to time-to-treatment delay. Those investigations are mandatory before specific, highly specialized therapeutic measures are taken (surgery, thrombolysis *etc*.). There are, however, therapeutic steps that can be performed earlier, on site, in the ambulance or in the emergency unit, such as lowering the blood pressure or the reversal of the anticoagulant therapy in case of intracerebral haemorrhage (ICH) and the pre-notification of the stroke unit for IS 2. There is clearly a need for a diagnostic test to be performed in the near-patient environment that could provide early warning as of what type of brain lesion is a stroke patient experiencing 3.

By cerebral imaging, an ICH is readily discernible; however, it is a different story with cerebral ischaemia. The therapeutic window is already closing when a diagnosis of acute ischaemic stroke (IS) is made with certainty. The question of what to do in the pre-hospital setting during the acute phase of the stroke is still unanswered: should the thrombolytic therapy be instituted immediately (without having diagnostic certainty, as ischaemia is visible with cerebral CT in the first 3 hrs after onset in only a third of the cases 4) or should the diagnostic confirmation be obtained first? Acting quickly and without diagnostic certainty could reverse the ischaemic process, but runs the risk of treating a patient who has no stroke (the neurological deficit may be caused by stroke mimics such as epileptic seizures or cerebral tumours) or who is at risk for a haemorrhagic transformation. Conversely, the certainty of an ischaemic process within the cerebral tissue leaves us with no alternative other than to take account of the damage and offer supportive treatment.

We are now able to offer comprehensive medical support for a patient after an acute stroke and to perform rehabilitation after the damage is done. Despite this, there can still be little chance for complete recovery. The sequels that exist after stroke can impose a huge financial burden on society and lead to hard-to-assess personal suffering 5. The only way of moving forward is to design new methods to diagnose IS within the therapeutic window or in the sub-acute phase. They allow the rapid identification of the correct therapeutic path. Such an opportunity seems to be offered by neuronal biomarkers 6.

Neuronal biomarkers are substances found in the neural tissue and are released into the blood stream after a neuronal injury, or more precisely after the disruption of the blood–brain barrier. If their blood levels correlate with the type and extent of the neural damage, it may be possible to supplement diagnostic capabilities, offering the best therapy as early as possible for patients in need 7.

Several neuronal proteins have been studied as would-be neuronal biomarkers and a limited number of substances are constantly cited as having significance for the diagnosis of stroke, but no consensus has been reached regarding their use in the clinic 8,9. Earlier reports state that some substances have certain specificity for ischaemic and haemorrhagic stroke. Glial fibrillary acidic protein (GFAP) is a biomarker candidate that appears to be indicative of ICH, while antibodies against *N*-methyl-d-aspartate receptor subunit NR2 (NR2 R_NMDA_) appear to be more specific for an ischaemic event 10,11. Nevertheless, neither marker alone appears to be sensitive and specific enough for clinical decision making. The GFAP has been recently investigated as a biomarker for ICH. It has been shown that it is released rapidly in acute ICH (the serum level being correlated with the size of the haematoma), whereas, a more delayed kinetic is expected in case of an ischaemic lesion. A GFAP cut-off of 0.29 μg/l provided diagnostic sensitivity of 84.2% and specificity of 96.3% for differentiating ICH from IS [Foerch 2012]. However, normal serum levels of GFAP cannot rule out ICH. There is an agreement among neurologists that a single biomarker cannot provide enough information and that a panel of substances should be used instead 12.

In this study, we wanted to determine whether GFAP and antibodies against NR2 R_NMDA_ could together discriminate between ICH and IS. This information could be used not only to efficiently diagnose individuals to optimize the triage and management of patients, but also to identify different physiological pathways that could reveal promising targets for sub-acute therapy to improve long-term outcome.

## Materials and methods

### Patient selection

Between December 2010 and November 2011 we enroled consecutive stroke patients and healthy controls in a prospective manner. We included in the study, patients with symptoms suspicious for acute stroke that were evaluated in the Emergency Department of our Hospital. The informed consent was obtained from study participants or their legal designates. The inclusion criteria were: time window between symptom onset and hospital admission <12 hrs, presence of hemiparesis at hospital admission and presence of clinical signs of hemispheric involvement. We used the following exclusion criteria: stroke patients presenting later than 12 hrs after the onset of the neurologic deficit, history of tumoural or traumatic lesions of the brain and a history of stroke within the 4 weeks prior to enrolment. The type and extent of the brain lesion (ICH or IS) were established using brain CT scan and the final diagnosis was established before discharge. The healthy controls were selected among the relatives of the patients; we included men and women with no known neurological diseases. The study protocol has received approval by the ethical board of our university.

### Sample collection

Samples of 5.5 ml of venous blood were taken into vacuum tubes with no additives from each patient at 12, 24, 48 and 72 hrs and 1 and 2 weeks after the onset of symptoms. For the healthy controls we assessed the blood levels of the neuronal biomarkers only once, at inclusion. Samples were centrifuged immediately at 1300 × g for 5 min. at 4°C, and the separated serum was stored at −80°C for further processing.

### GFAP measurement

Serum GFAP levels were assessed using a commercial human GFAP ELISA kit (BioVendor, Heidelberg, Germany) following the manufacturer’s instructions. Samples in duplicate were diluted at 1:3 before analysis, and 100 μl was added to the microplate wells. Quality controls and increasing concentrations of standards were done for obtaining the calibration curve. After 2 hrs of incubation at room temperature, 100 μl biotin-labelled anti-GFAP-antibody solution was added to each well, followed by a 1 hr incubation period. The detection step consisted of incubation with HRP (horse radish peroxidase)-conjugate for 1 hr followed by pipetting in 100 μl 3,3′,5,5′-tetramethyl benzidine (TMB) substrate for colorimetric detection. After stopping the colour reaction, the plate was immediately read at 450 nm with a Sunrise Tecan microplate reader equipped with the acquisition software Magellan 3. The entire experiment was performed at room temperature and between each step a washing procedure was applied (4 cycles of 400 μl washing solution/well). Serum values (ng/ml) were extracted from the standard curve.

### Detection of serum antibodies to NR2 subunit of NMDA receptor

The Gold Dot NR2 Antibody Assay kit (CIS Biotech, Inc., Atlanta, GA, USA) is an ELISA method designed to quantify the serum levels of antibodies directed against NR2 peptide, an NMDA receptor subunit. We analysed serum samples according to the manufacturer’s manual. 100 μl of diluted patient and control serum samples (1:50), calibrators, positive control and negative controls were added to a microtiter plate and incubated for 30 min. at 37°C on a shaker to allow the antibody to react with the NR2 peptide coated on a solid phase of the microplate. After washing for three times with 200 μl of working solution (5 min. during each wash at 37°C, on a shaker), 100 μl of protein A-HRP was added to each well and incubated for 30 min. at 37°C on a plate shaker. Another washing step was performed as described above followed by a onetime washing step with 200 μl of deionized water for 1 min. on shaker. After pipetting 100 μl of the TMB substrate, the microplate was gently shaken in darkness for 10 min. at room temperature to achieve colour development. The enzymatic reaction was stopped with acid solution and read after 10 min. using a Bioteck Synergy 2 microplate reader at 450 nm/630 nm. A standard calibration curve was generated and corresponding values in ng/ml were determined. Assays were done in duplicate.

### Statistical analysis

We used Exact Wilcoxon–Mann–Whitney *U*-test to compare the average values of GFAP and of antibodies against NR2 R_NMDA_ from patients who had IS or ICH. To discriminate between ischaemic and haemorrhagic stroke we used binary logit regression. We associated the area under the receiver operating characteristic (AUROC) curve to the regression.

## Results

Seventy-two stroke patients were enroled (49 patients having IS, 23 having ICH). Table1 displays the baseline variables of the study population.

**Table 1:** Baseline characteristics of the study population

	Ischemic stroke	Intracerebral haemorrhage	Controls
*N*	49	23	52
Mean age (years) (SD, interval)	71.8 (4.38, 54–80)	63.3 (4.51, 59–74)	68.9 (5.91, 53–81)
Men, %	79.5%	73.9%	75%
NHISS at admission	13.3 (10, 1–31)	18 (5.9, 6–28)	
NHISS at 2 weeks (SD, interval)	13.7 (11.7, 1–36)	15.8 (6.3, 4–26)	
Aetiology of IS (*N*, %)			
Large artery disease	9 (18.4%)		
Embolic stroke	28 (57.1%)		
Small artery disease	7 (14.3%)		
Unknown	5 (10.2%)		
Size of the ischemic brain lesion (*N*, %)			
Less than one-third of middle cerebral artery territory	19 (38.8%)		
One-third to two-thirds of middle cerebral artery territory	7 (14.3%)		
More than two-thirds of middle cerebral artery territory	13 (26.5%)		
Vertebro-basilar artery territory	10 (20.4%)		
Size of the haematoma (*N*, %)			
1–20 ml	9 (39.1%)		
21–40 ml	8 (34.8%)		
41–60 ml	4 (17.4%)		
>60 ml	2 (8.7%)		

**Figure 1:**
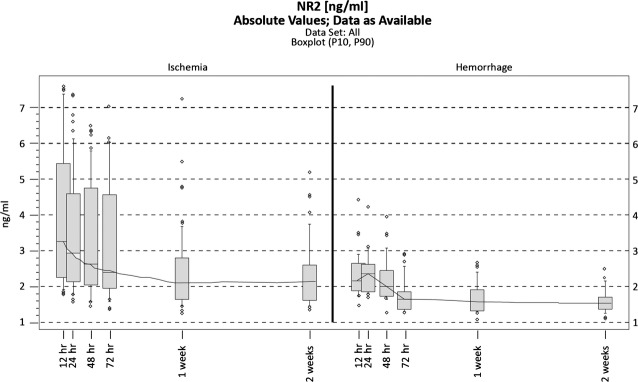
Antibodies against NR2 R_NMDA_ over time in ischemic and haemorrhagic stroke patients (box plots). The boundaries of the box indicate the 25th and 75th percentile, and the line within the box marks the median. Whiskers above and below the box indicate the 90th and 10th percentiles.

**Figure 2:**
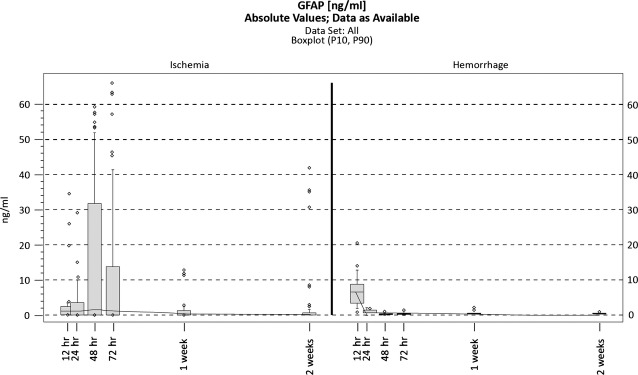
Antibodies against NR2 R_NMDA_ levels may be indicative of IS at all time-points.

**Figure 3:**
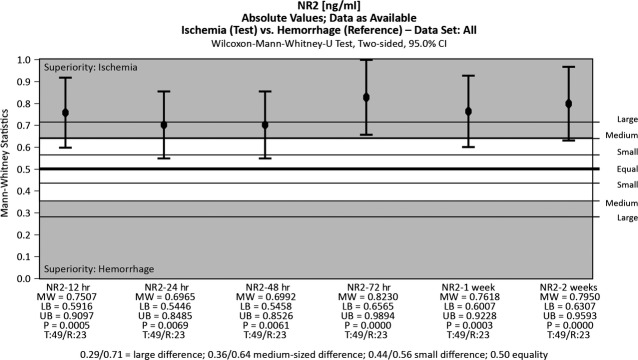
Glial fibrillary acidic protein (GFAP) over time in ischemic and haemorrhagic stroke patients (box plots).

The two neuronal biomarkers were assessed in serum samples taken at the above-mentioned time-points and the results are presented in Figures1 and 2. The serum levels of the antibodies against NR2 R_NMDA_ were significantly higher in cases of IS compared to ICH. They peaked at 12 hrs (IS) and 24 hrs (ICH) after the onset of the neurological deficit and then slowly declined; this behaviour is similar in cases of IS and ICH, but the absolute values significantly differed, as seen in Figure3.

**Figure 4:**
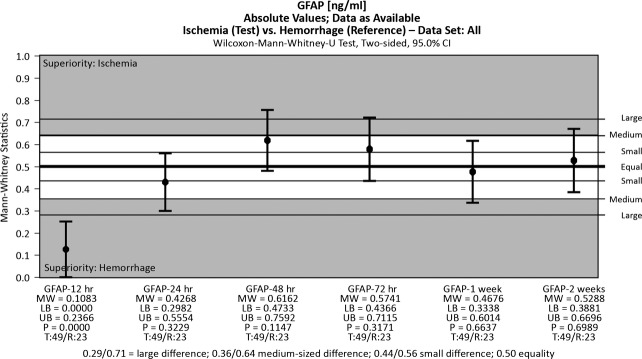
Glial fibrillary acidic protein (GFAP) may be indicative of intracerebral haemorrhage (ICH) at 12 hrs after onset.

Glial fibrillary acidic protein levels have markedly different patterns of variation in IS and ICH. During IS the blood levels were low in the first hours after onset and then rose and peaked at 48 hrs. In cases of ICH, they peaked within the first 12 hrs, and then the serum concentration fell rapidly to normal levels, as seen in Figure4.

The levels of GFAP and of antibodies against NR2 R_NMDA_ assessed within 12 hrs after the onset of symptoms could be used to identify the type of stroke (IS or ICH) with a discriminant function – binary logit regression (see Table2). By using binary logit regression we can estimate the probability of ischaemia or haemorrhage in the case of a stroke patient with the formula presented below, knowing that Prob(*ICH* = 1) = 1 − Prob(IS = 1).

**Table 2:** Binary Logit Regression – discriminant function between ischemic and haemorrhagic stroke

	12 hrs		24 hrs		48 hrs	
	Coef.	*P*-value	Coef.	*P*-value	Coef.	*P*-value
GFAP_12 hrs	−0.236	0.001	−0.002	0.981	0.303	0.280
NR2_12 hrs	1.954	0.005	0.833	0.020	0.195	0.682
Constant	−3.353	0.023	−1.564	0.081	−0.318	0.756



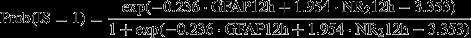


**Figure 5:**
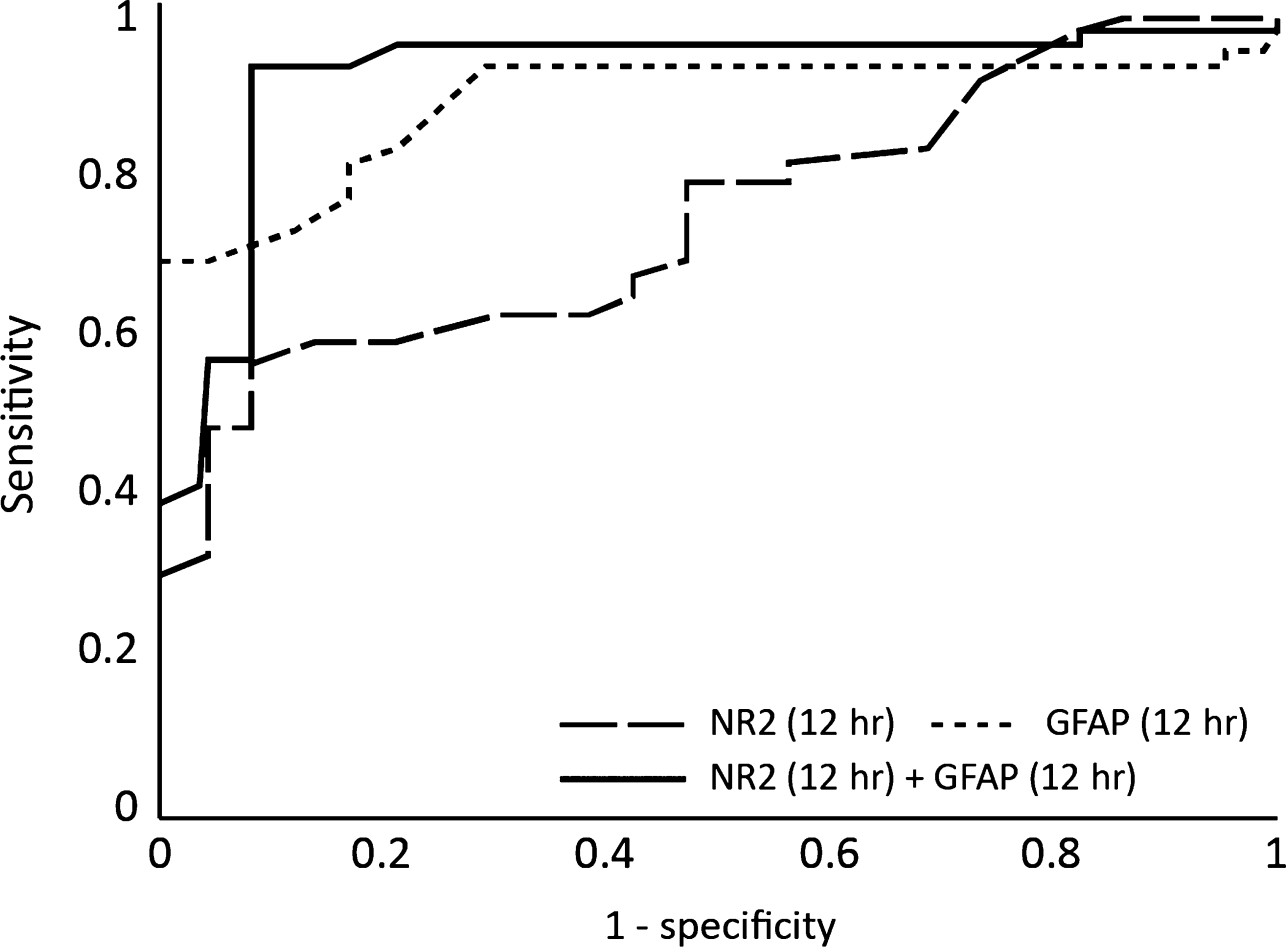
The ROC analysis after Binary Logit Model for glial fibrillary acidic protein (GFAP) and antibodies against NR2 R_NMDA_ at 12 hrs after the onset.

The Receiver operating characteristic (ROC) analysis based on the values derived from the presented formula indicates that together, GFAP and NR2 R_NMDA_ can discriminate between ischaemic and haemorrhagic stroke at 12 hrs after onset with a sensitivity and specificity of 94% and 91%, respectively, as shown in Figure5.

**Figure 6:**
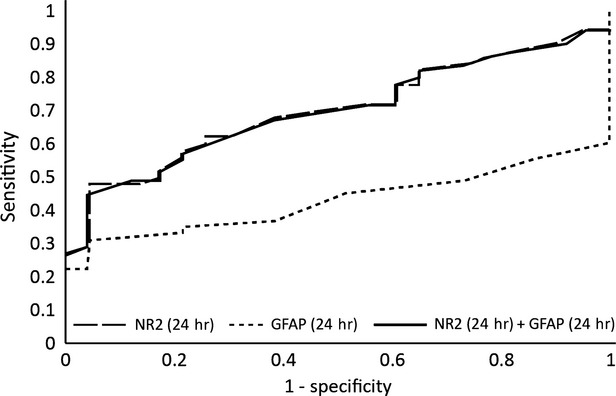
The ROC analysis after Binary Logit Model for glial fibrillary acidic protein (GFAP) and antibodies against NR2 R_NMDA_ at 24 hrs after the onset.

**Figure 7:**
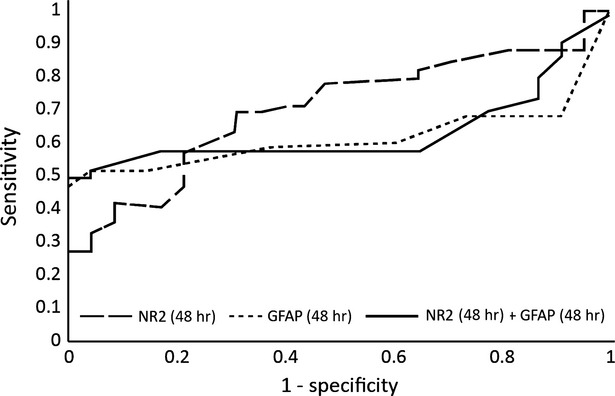
The ROC analysis after Binary Logit Model for glial fibrillary acidic protein (GFAP) and antibodies against NR2 R_NMDA_ at 48 hrs after the onset.

At 24 hrs the same biomarkers are no longer relevant, as the percentage of correct predictions is insufficient (Fig.6). Furthermore, the AUROC for both markers is almost identical as the one for NR2, so GFAP has no significant impact after this time-point (Fig.7).

## Discussion

A diagnostic approach based on biomarker testing has potential advantages, including cost-effectiveness and widespread availability. Performing those tests in the primary care units would facilitate rapid patient transport to a hospital with an integrated stroke team. In this study, we found that the neuronal biomarkers GFAP and NR2 R_NMDA_ are able to distinguish IS from ICH with 94% sensitivity and 91% specificity at 12 hrs after onset of symptoms.

The role that neuronal biomarkers should play in the diagnostic workup of a stroke patient is still not well-established. The occurrence of neuronal proteins in the blood stream implies two necessary conditions: a source and an avenue, *i.e*. neuronal death, disorganization of the extracellular matrix and increased permeability of the blood–brain barrier. Both ischaemic and the haemorrhagic stroke lead to brain cell death and barrier dysfunction. However, there are subtle differences between them, both in the way they cause cell death and in the way they increase barrier permeability, and those differences could provide the opportunity for a differential diagnosis.

During ischaemic episodes, sudden cerebral tissue hypoperfusion initiates a series of events known as the ischaemic cascade. These events include cellular energetic failure, excitotoxicity, oxidative stress, erratic enzymatic activation, microvascular injury and cell death 13. Disruption of the blood–brain barrier in IS is a biphasic event and is influenced by the reperfusion that occurs after injury. Within the first 24 hrs, there is increased permeability of the blood–brain barrier as a result of extracellular enzymatic activation, and further damage occurs 48–72 hrs after the infarction 14. In the case of an ICH, the neural damage is caused initially by the compressive effect (thus the damage depends greatly on the volume and expansion rate of the haematoma), and the blood–brain barrier remains intact 15. Within the first hours after onset, varying degrees of oedema appear in the surrounding tissue and osmotically active substances are released. The systemic inflammatory response and activation of thrombolysis within the cerebral clot, coupled with the neuronal toxicity of haemoglobin (events occurring 2–3 days after the hemorrhagic event), complete the spectrum of factors that cause neuronal death and irreversible brain damage. The brain–blood barrier remains intact longer during an IS, thus brain-specific proteins appear in the blood stream 8–12 hrs after onset. Other authors have reported the reverse order, with the barrier being destroyed sooner during a haemorrhagic stroke than during an IS 16. Despite these contradictions, knowing that the brain tissue damage is caused through different mechanisms in the two types of stroke offers the potential to discriminate between types of stroke through the use of circulating neuronal biomarkers.

Many molecular biomarkers that have been implicated in the neurovascular injury cascade after stroke have been studied in recent years with regard to their ability to predict the type of stroke (ischaemic or haemorrhagic), including inflammatory mediators such as monocyte chemotactic protein-1, matrix metalloproteinase-9, tissue inhibitor of matrix metalloproteinase-1, interleukin-6, C-reactive protein, ficolin-3 and the brain damage markers S100B and proenkephalin neuropeptide 17. Other studied markers include B-type neurotrophic growth factor, von Willebrand factor, GFAP and NMDA-R fragments 18–20. The diagnostic significance and potential clinical utility of these biomarkers remains to be validated.

Glial fibrillary acidic protein peaks early during haemorrhagic brain lesions and late (at significantly higher levels) in ischaemic events. Glial fibrillary acidic protein was recently identified as a biomarker that is indicative of ICH in the acute phase of stroke. In a prospective study that included 93 patients who had an IS and 42 patients who had an ICH, within 6 hrs after the onset of symptoms, GFAP was detectable in the serum of 81% of ICH patients, but only in the serum of 5% of IS patients 10. The average GFAP serum concentration was significantly higher in patients who had an ICH. A cut-off point of 2.9 ng/ml was found to provide a sensitivity of 79% and a specificity of 98% for the differentiation of ICH from IS. In our study, GFAP was a good marker for identifying haemorrhagic stroke within 12 hrs after the onset of symptoms, a high blood level being indicative of a haemorrhagic event. The maximum level was seen at 12 hrs after an ICH and at 48 hrs after an IS. At 12 hrs after the onset of symptoms the sensitivity and specificity for discriminating ischaemic and haemorrhagic events were 94% and 69%, respectively.

The fragmentation of the NR2 subunit into the peptides NR2A and NR2B is caused by ischaemia or neurotoxicity. The NMDA receptor peptide is thus an indicator of glutamate excitotoxicity associated with acute stroke pathophysiology and can be used for the diagnosis of acute IS (in preliminary clinical studies, the biomarker had a sensitivity of 97% and a specificity of 98%) 21. In our study, the antibodies against NR2 R_NMDA_ had different patterns of variation during IS and ICH, as has been previously described 22. This biomarker was very good at discriminating between stroke patients and healthy controls at any time-point (specificity 96% at 12 hrs and 100% at 24 and 48 hrs after the onset of symptoms). When used to differentiate IS from ICH, the results did not produce the same significance at 12 hrs after stroke.

Used independently, the above-mentioned biomarkers are insufficient to differentiate between ischaemic and haemorrhagic stroke with any accuracy. However, we demonstrated that the sensitivity for GFAP alone is 94%. The additional predictive ability comes from an increase in sensitivity when adding in NR2, so that by using the levels of GFAP and of NR2 R_NMDA_ antibodies, at 12 hrs after onset, we can identify with a very good sensitivity and specificity (94% and 91%, respectively) the type of stroke. This time-point is significant for a number of reasons. First, the anatomical brain lesion is usually not fully established prior to 12–24 hrs after the onset of symptoms 23. Within this period, the penumbral tissue is potentially salvageable by therapeutic intervention, but only if driven by thorough knowledge of the nature of the brain lesion (ischaemic or haemorrhagic). Unfortunately, the type of stroke is not always identified by early brain imaging (the limitations of CT to show acute ischaemia in the posterior fossa are well known,), and access to brain CT scan is not always available 24. Wardlaw demonstrated that access to a brain CT scan was distributed unevenly in Scotland, with only 65% of patients benefitting from a CT scan within the first 48 hrs after a stroke. This figure rises to 100% only after 7 days for hospital-admitted patients 25. However, an important conclusion of his study was that the best strategy for approaching stroke patients (both in terms of limiting sequelae and long-term costs) was to invest more in the acute phase (*i.e*. scan all patients at the earliest moment). Given this line of reasoning, any other tests that would increase our diagnostic capabilities are welcome.

There is a rigid 3-hr limit imposed for the initiation of thrombolytic therapy in IS imposed by the limitations of CT brain imaging. Several reports have suggested that a preselection of patients using other diagnostic tools, such as multiparametric MRI protocols that use diffusion-weighted and perfusion imaging can identify patients who have had a stroke and who may benefit from intravenous thrombolysis within and beyond 3 hrs after the onset of stroke symptoms. Thrombolysis therapy based on MRI seems safer and potentially more efficacious than standard CT-based thrombolysis 26. Therefore, another reason for trying to establish the aetiology of stroke beyond this limit is to extend the time interval in which thrombolytic therapy could be used. The neuronal biomarkers would be very valuable for increasing clinical confidence and diagnostic accuracy for therapeutic decision making.

Vascular cognitive impairment (VCI) is now the preferred term to describe the spectrum of cognitive impairments that are associated with frank stroke, vascular brain injury or subclinical disease. This impairment imposes a huge burden upon both the healthcare system and the family of the patients. The prevalence of post-stroke dementia varies in relation to the interval after stroke, location and size of the brain lesion. The prevalence of dementia is 30% immediately after stroke, and the incidence of new-onset dementia increases from 7% after 1 year to 48% after 25 years 27. To date, there is no specific treatment for VCI approved by regulatory institutions worldwide. These figures underline the need for accurate diagnosis in the acute and subacute phase of stroke because aggressive treatment in this phase with control of other vascular risk factors could lower the prevalence of VCI 28.

Targeted therapies for stroke are a new, promising field of research. Using nanotechnology, researchers have recently proved in experimental models that certain drugs can be delivered precisely to the injured areas of the brain, thus maximizing their therapeutic effect and minimizing their side effects 29. Tissue factor is a membrane glycoprotein and the primary initiator of coagulation. It is over-expressed in areas of brain microvascular endothelial cells injury and triggers a cascade of molecular events that leads to coagulation. Expression of its gene can be reduced by an RNA-induced silencing complex based on small interfering RNA. It has been recently proven that it can be delivered precisely to the area of the brain endothelial lesion by modified poly(lactic-co-glycolic acid) (PLGA) nanoparticles coupled with the Enhanced Green Fluorescent Protein-Epidermal Growth Factor (EGFP-EGF) fusion protein 30.

The main limitation we see in using biomarkers in stroke patients (and the one that precluded their use outside clinical trials) is the lack of commercially available rapid diagnostic tests that could be easily performed in the Emergency Department or even in a pre-hospital setting. However, once their importance is convincingly established, it is likely that the technology will follow. The use of biomarkers is routine nowadays in other fields of emergency medicine, such as in the workup of chest pain (for instance B-type natriuretic peptide).

We feel that there are several limitations with regard to the study design, such as the small number of individuals, the potential for lack of generalizability and the lack of an earlier time-point, within the first 3 hrs after onset. The latter was not included in the protocol because of technical and logistical issues, as the clinical path assigned to a patient with suspected stroke did not always allow the study team to perform the study-related tests. A 3 hrs time-point could do an even better job of discriminating between IS and ICH by placing the diagnosis during the therapeutic window. This can be an area of future study, prior to establishing a biomarker-based diagnostic protocol. Nevertheless, by describing the kinetics of both GFAP and antibodies against NR2 R_NMDA_ in the following 2 weeks after stroke we demonstrated that used together, the biomarkers can discriminate between the two types of lesions even after the first 12 hrs. This time limit after stroke was established earlier for the GFAP use and is important mostly for the cases in which we do not know the time-point for the onset of the symptoms (‘wake-up strokes’) 31.

The patients who had other neurological factors (*e.g*. traumatic brain injury, epileptic seizures or cerebral tumours) were not included as controls; thus, no statement can be made about the ability of these biomarkers to differentiate between stroke and these other neurological disorders with similar clinical presentation.

We conclude that the levels of GFAP and of antibodies against NR2 R_NMDA_ can discriminate between ICH and IS in acute stroke patients. When used together, these two biomarkers offer a very good sensitivity and specificity in differentiating between the two types of stroke at 12 hrs after onset. If the model described in this paper were to be adopted in the pre-hospital setting, it could potentially improve patient outcome after stroke by optimizing the triage and thus allowing clinicians to rapidly determine the appropriate clinical care path. Nevertheless, we consider that this model should be validated prospectively in an independent cohort.
